# Treasure of the Past VIII: Molecular Basis of Flame Inhibition*

**DOI:** 10.6028/jres.106.034

**Published:** 2001-08-01

**Authors:** J. W. Hastie

**Affiliations:** Institute for Materials Research, National Bureau of Standards, Washington, D.C. 20234

**Keywords:** Fire retardants flame inhibition, flames

## Abstract

The role played by inorganic chemical additives in fire retardancy and flame inhibition is considered. Particular attention is given to the molecular level aspects of commercially important systems containing compounds of antimony, halogens, and phosphorus. The flame inhibiting function of metal containing additives is also discussed.

## 1. Background

### 1.1. Introduction

It has long been recognized that the addition of chemical substances to combustible materials can considerably reduce the degree of flammability. In the specific case of textile fabrics, an extensive technology has developed through which a wide range of chemically distinct incorporants can be utilized to retard flammability while other desirable properties such as permanence of treatment, appearance, wear resistance, and hand texture are retained [1–6]^1^.

A well-developed empiricism has been elaborated which relates retardant characteristics such as elemental composition, chemical structure, and degree of loading to the reduction of flammability. The literature of this and related fields offers considerable deductive insight into the mechanisms by which fire retardants function. However, it is apparent, as Lyons [2] has stated, that:

“Although much can be deduced from the results of applied studies in the literature, there have been relatively few experiments conducted specifically to determine the mechanisms of retardance. At this stage, most of what’ can be said is highly speculative.”

While the undoubted successes of applied chemistry in flame retardancy are clearly apparent, it has become increasingly important, as requirements for reducing the flammability of combustible materials become more stringent, to develop a detailed understanding at the molecular level of the mechanisms by which flame retardants operate. Knowledge of these essential chemical processes can, for example, permit more sophisticated design of retardants for particular applications and perhaps define the performance limits to be expected of particular retardant systems under service or test conditions. Candidate retardant materials can be selected more efficiently for screening if their chemistry can be related to the fundamental basis of retardant action. Retardants can be formulated that optimize performance and minimize undesirable side effects such as formation of smoke and toxic reaction products.

The combustion of a fabric, or organic polymer, involves a preflame thermal degradation of the material, resulting in the evolution of flammable volatiles, and the gas phase pyrolysis or oxidation of the evolved gases. Chemical fire retardants may operate in the solid phase to modify the thermal degradation process or in the gas phase by yielding volatile products that inhibit the flame reactions. A given retardant formulation may, of course, operate in both modes.

For purposes of discussion the general subject of fire chemistry may be usefully visualized in terms of the contributing phenomena of ignition, propagation, and cessation, as indicated by table 1. Note that a fundamental description of fire propagation and cessation (i.e., inhibition or extinction) and the production of toxic gases and smoke requires an understanding of flame chemistry and at a molecular level.

The detailed mechanism of solid-phase retardant action is a complex problem of organic reactions in the solid state. For the degradation of cellulose, for example, both free radical reactions and acid-catalyzed rearrangement pathways have been suggested. Speculations have been offered concerning the role of retardants such as phosphorus compounds in modifying the degradation [7]. This aspect of fire retardancy will not be considered in the present discussion, which focuses attention on the gas-phase mode of flame inhibition.

The purpose of this paper, then, is to consider the molecular level mechanistic aspects of the gas-phase mode of fire retardancy. In dealing with this problem at the molecular level it will become apparent that there are fundamental connections in the details of the mechanisms underlying the phenomena of flame inhibition, fire extinction and smoke production. It is axiomatic that the question of toxic gas production is also related to these phenomena although this will not be pursued here.

### 1.2. Chemical Aspects of Flame Inhibition

It is now recognized that the inhibition or extinguishment of flames can more effectively be achieved by chemical, rather than physical, means. However in practical fire retardancy situations, both physical and chemical effects operate. For the present discussion we will be concerned only with the chemical aspects of flame inhibition.

A number of reviews and surveys, concerned primarily with the chemical aspects of flame inhibition, have been made [8–13]. Discussions of the relationship of flame inhibition chemistry to fire extinguishment or fire proofing in general may be found in a number of comprehensive literature sources [2–4, 14].

In practical systems the manner in which the additives are released to the flame, or potential flame, system is of importance. One of the theories based on empirical data requires the additive to be “at the right place at the right time.” Thus, to some extent the protection of flammable substrates requires that the vapor release properties of the inhibitor be related to the fuel release properties of the flammable substrate. As an example one may cite the antimony oxide — organic halogen formulations used to reduce the flammability of organic polymers where the antimony-halogen component is released at about the same time as the polymer decomposes (e.g., see Pitts [15]).

The considerable variety of chemical systems used to impart fire retardancy to materials has been documented by Lyons [2]. A satisfactory theory of flame inhibition has not yet been derived, although it is generally agreed that the additives presumably interfere with the concentration of the flame propagating radicals H, OH, O, and perhaps CH_3_ and HO_2_. The influence of additives on flame speed, also known as burning velocity, is particularly revealing in that only small amounts of additives are required to strongly affect flame propagation. It is also apparent that the inhibiting action of additives, such as halogens, cannot be explained merely in terms of a reduction of the equilibrium concentration of radicals due to hydrogen halide formation. Evidently the function of the additive is catalytic and flame inhibition is primarily a kinetic phenomenon [9].

## 2. Retardancy Chemistry Relating to Pyrolyzing Substrates

A microscopic view of an idealized burning system is expressed by the schematic of table 2. It is considered that the function of substrate additives is to provide a source of “radical traps” to the gas phase where they may function either in the preflame or reaction zone regions of the flame.

The usual macroscopic criteria for defining a gas phase, as opposed to a condensed phase, mode of retardancy activity are summarized in table 3.

### 2.1. The Antimony Oxide—Halogen Example

The well-known antimony oxide — halogen fire retardent system, where the halogen is an organic chloride or bromide, serves as an example of the application of such criteria and the underlying molecular aspects involved.

Table 4 summarizes the main reasons why an intensive consideration of this system appears to be warranted.

Among practicing fire retardancy chemists, synergism is one of the more desirable goals. The achievement of a synergism allows the utilization of flame inhibiting additives at lower concentrations, and the antimony oxide — halogen combination represents one of the more important synergistic fire proofing combinations. The synergistic nature of this system is exemplified by the fact that a fireproofed epoxy system containing 15 percent Br can be replaced by a combination of 5 percent Br + 3 percent Sb_4_O_6_. Thus the quantity of halogen needed is reduced by the presence of Sb_4_O_6_ (i.e.2Sb_2_O_3_).

The combination of antimony(III) oxide, Sb_2_O_3_, with a halogen source, most commonly a halogenated organic material, is a well known example of a synergistic flame retardant system [16]. The ability of antimony to enhance the effectiveness of halogen-based flame retardants was first demonstrated for cellulosic fabrics treated with chlorinated paraffins and antimony trioxide [17]; the synergism has also been shown, inter alia, in polyester resins [18–20], polystyrene resins [21], and polyolefins [22]. Use of antimony compounds in conjunction with halogenated flame retardants is also documented for polyurethanes, polyacrylonitrile, and polyamides [2, 16]. Despite the availability of some alternative materials and uncertainties in supply leading to a continuing search for other substitutes, it is likely that the use of antimony chemicals for this purpose will increase significantly over the next few years, particularly in plastics [23].

This fire retardant system has been in use for more than 30 years. However as recently as 1967 no satisfactory theory had been suggested to explain the synergism between halogen and antimony compounds in imparting fire retardancy to polymer compositions [4].

The recent literature contains numerous speculations as to the mechanism, including the following:

— formation in situ of antimony chloride which may react with cellulose to alter the course of thermal decomposition and/or form a “heavy vapor tending to extinguish the flame” [24].
— formation in the flame of nonvolatile, antimony-containing solid or liquid particles whose surface provides a site for dissipation of energy with resulting modification of the flame chemistry (e.g., formation of HO_2_ rather than HO — wall effect) [25].
— “formation of an antimony oxygen halogen intermediate compound which increases the presence of halogen radicals with resulting interference in the free radical mechanism of the flame propagation” [21].
— “formation of antimony halides or oxyhalides which may act by blanketing the flame” [26].
—”… the oxygen inhibiting effect of the heavy vapors of antimony chloride or oxychloride in addition to the physical inhibition of the oxidation chain reaction (wall effect) and chemical inhibition by chlorine” [3].

It is known that much of the antimony is vaporized during burning [27] or char formation [24]. The ignition behavior of polyester resins inhibited by antimony-halogen systems has been considered to indicate the likelihood of gas phase inhibition [19, 20]. Further, the observation [27] that SD_2_O_3_ inhibits the burning of chlorinated polyethylene in oxygen, but not in nitrous oxide, suggests inhibition of gas phase reactions specific to the fuel-oxidizer system. On the other hand, some involvement of antimony in decomposition of the solid phase is indicated by the fact that char formation may be enhanced in antimony-containing systems [2, 4].

The synergistic action clearly involves interaction of Sb_2_O_3_ with a decomposition product of the halogenated material, presumably HCl. Optimum conditions for retardancy depend on the gross ratio of antimony to chlorine and on the ease of decomposition of the chlorinated species [2]. In an extensive series of studies on cotton fabric, optimum weight ratios of chlorinated paraffin to antimony oxide were found to be somewhat in excess of those required for conversion to SbOCl [17]. A study of the flammability of polyethylene by the oxygen index method, however, indicated a maximum effect with six chlorine atoms per atom of antimony (with one Sb present per 100 C_2_ units). At this level of antimony loading, a chlorine: antimony ratio of 2 was insufficient to produce the optimum effect [22].

Antimony oxychloride, SbOCl, is itself effective as a flame retardant [15] and has been proposed as the product responsible for flame retardant effects in cellulose systems [17]. The thermal decomposition of this substance has been studied by Belluomini et al. [28], and more recently by Pitts et al. [29], who also investigated its flame retardancy effect in flexible urethane foam. By trapping the gaseous products, the latter workers deduced SbCl_3_ to be the only volatile species. On the other hand, an early literature report [30] suggests that SbOCl vaporizes an appreciable amount of SbOCl species.

A summary of some typical macroscopic observations relating to antimony-halogen action is given in table 5. From these results it is apparent that the retardancy mechanism is of the gas-phase type. More recent mass spectrometric experiments using the Kundsen effusion method convincingly support this view [32]. Basically, the experimental procedure involves the passing of HCl gas over solid Sb_2_O_3_, contained in a Knudsen cell, and the composition of the effusing molecular beam is determined by a line-of-sight mass spectrometric analysis. The principal observations from this molecular level study are listed in table 6.

**Figure 1:**
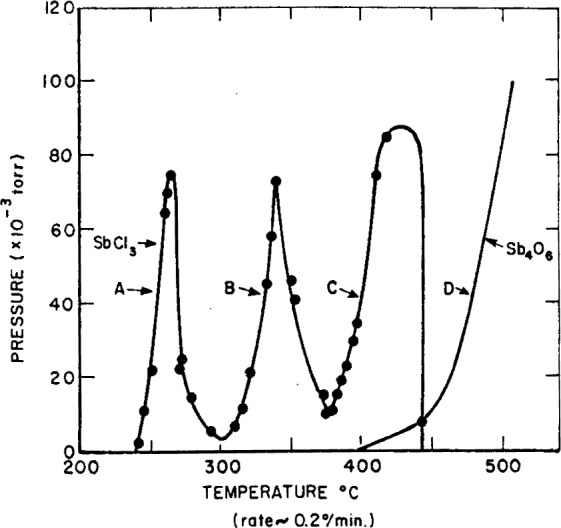
Pressure variation of the vapor species SbCl_3_ (curves A, B, and C) and Sb_4_O_6_ (curve D) as a function of temperature and time using solid SbOCl as a source and a heating rate of about 0.2 C° min^−1^.

**Figure 2:**
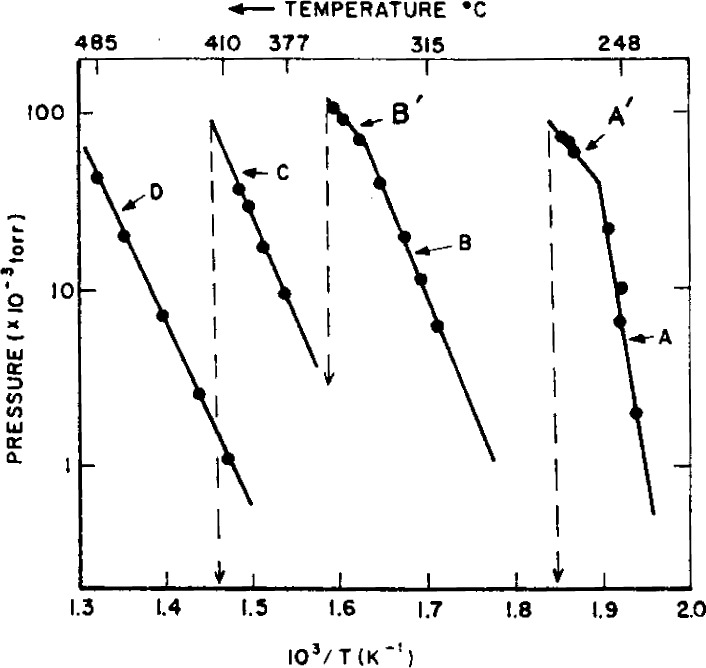
Clausius-Clapeyron log p versus 1/T plots of the vapor species generated by SbOCl as given by figure 1.

**Figure 3:**
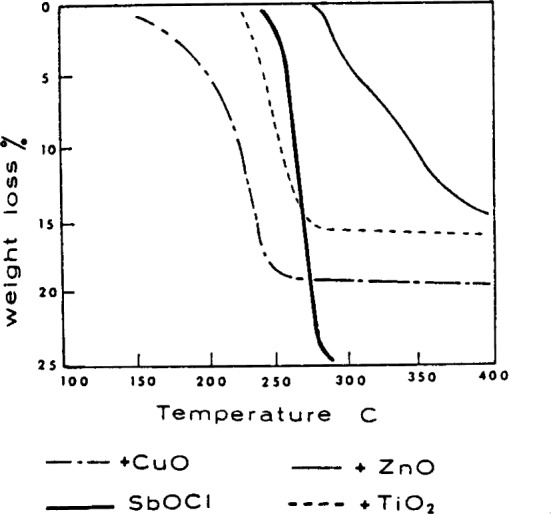
Thermogravimetric analysis curves of antimony oxychloride-metal oxide mixtures, from Pitts [15].

For conditions where SbOCl solid is present an un-usual mode of release of antimony and halogen to the vapor phase occurs, as indicated by the results shown in figure 1. These results were obtained under Knudsen effusion conditions where normally equilibrium is achieved and therefore Clausius-Clapeyron-type plots, as shown in figure 2, should yield lines with slopes proportional to the reaction enthalpy changes. The results may be interpreted to indicate that only part of the decomposition regime achieves equilibrium, i.e., Segments *A’* and *B’* of figure 2. For the most part, e.g., segments *A*′ and *B*′, a considerable activation energy, of about 70 kcal mol^−1^, is required. It is suggested that this activation energy barrier, which most likely is a solid phase diffusion controlled process, is the reason for the observed action [15] of other metal oxides on the decomposition of SbOCl as shown by figure 3. Similarly the improved retardancy effect provided by a partial replacement of Sb_2_O_3_ with 2ZnO · 3B_2_O_3_ · 3.5H_2_O, also known as Firebrake ZB, may be due to a lowering of the activation energy barrier [33].

The substrate reactions in the antimony oxidehalogen system are summarized in table 7.

As the vapor pressure of SbCl_3_ is now known for the system HCl+SD_2_O_3_ (or SbOCl), one may derive the Langmuir rate of vaporization i.e.,

$$G=P/[17.14{\left(T/M\right)}^{1/2}]\;g/{\text{cm}}^{2}\;s$$

where *P* is in torr and *M* is the molecular weight. Typically, a temperature of 550 K and *P* ~ 10^−1^ torr would correspond to a value of *G* ≃ 10^−2^ g/cm^2^ s. At this rate the concentration of SbCl_3_ species entering the region of the flame is approximately calculated to be greater than the concentration of the propagating radicals OH, H, and O. It would therefore seem inefficient for a retarding system to provide vaporization rates substantially in excess of this value. However, the “normal” decomposition behavior of an oxychloride system would result in *G* increasing exponentially with temperature. This would result in the synergistic system being effective only over a very narrow temperature interval due to the rapid depletion of sample for pressures in considerable excess of 10^−1^ torr. The relevant point to be made regarding the SbOCl decomposition scheme, however, is that the decomposition rate does not increase monotonically with temperature (see fig. 1) due to the formation of relatively stable intermediate solid phases. In effect, for sufficiently slow heating rates, the SbOCl decomposition may be described as a “triple shot” fire extinguisher. For relatively high heating rates one would not resolve this “triple shot” effect; however, the average rate of vaporization would still be far less than that of a “normal” system.

Thus it appears that at least part of the synergism associated with this system derives from the ability of Sb_2_O_3_ to moderate the release of halogen to the gas phase and over a considerable temperature range. This effect has a parallel in the use of substrate free radical initiators, such as dicumyl peroxide [34], which are believed to delay the loss of halogen from the decomposing polymer.

### 2.2. The Triphenylphosphine Oxide Example

A second example of a fire retardant system where vapor phase processes form the basis for flame inhibition is the triphenylphosphine oxide-Nylon 6-polyethyleneterephthalate system (i.e., TPPO-Ny6-PET). The effect of small additions of TPPO and Nylon 6 on the flammability of the PET polyester is indicated in table 8. Macroscopic evidence for a vapor phase mode of inhibiting action has been obtained recently by Bostic and Barker [35].

**Figure 4:**
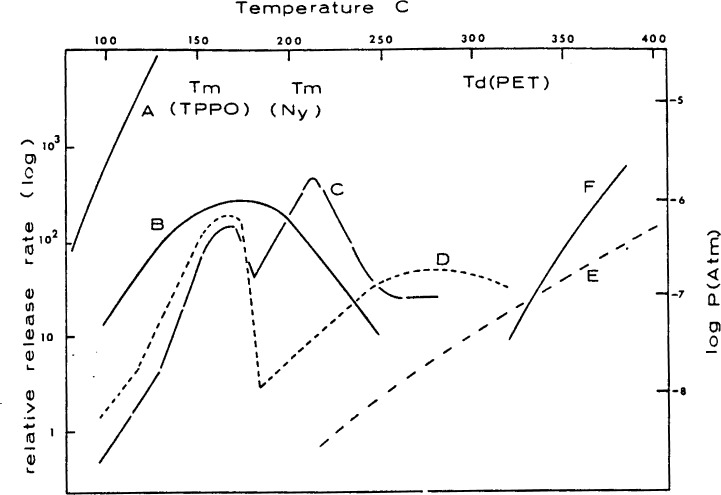
Vaporization rates of TPPO (except for case F) as a function of temperature for various triphenylphosphine oxide (TPPO)-Nylon 6 (Ny-6)-polyethyleneterephthalate (PET) systems: A is for pure TPPO solid, B is for 6.7 mol percent TPPO in Ny-6, C is for 1 percent TPPO in PET, D is for 1 percent TPPO + 1 percent Ny-6 in PET, E is for pure TPPO × 10^−6^ and F is for the caprolactam monomer vapor from Ny-6; Tm and Td indicate melting and decomposition temperatures respectively.

The results of recent mass spectrometric Knudsen reactor studies [36] are summarized in figure 4. The curves indicate a nonmonotonic dependence for the rate of TPPO release from PET substrates with increasing temperature which is reminiscent of the SbOCl system. However it is apparent from these results that this system is not as efficient as the antimony oxide-halogen system. That is, a significant fraction (~ 50%) of the TPPO retardant is lost from the substrate prior to pyrolysis of the PET or Nylon 6. The synergistic effect of the Nylon-6 component, as suggested by the observations in table 8, appears to be due to an increased retention of the TPPO so that it is available at a higher temperature.

Table 9 lists a suggested reaction sequence for the mechanism of release of TPPO from PET-Nylon 6 substrates. A more detailed account of these recent studies has been given elsewhere [36].

## 3. Molecular Nature of Flames and Their Inhibition

### 3.1. Nature of Flames

The following discussion considers the flame retarding action of inhibitors such as SbBr3, TPPO, and other additives or extinguishants of practical interest. However, in order to discuss mechanisms of flame inhibition it is first necessary to define the mechanisms of flame propagation. This involves consideration of the basic chemical and kinetic character of flames as summarized briefly in tables 10 and 11.

Examples of flame reaction mechanisms are given in table 12. It is generally agreed that the most important step in normal flame propagation is the chain branching reaction:

$$H+O_{2}=\text{OH}+O.$$

As with the previous discussion of substrate chemistry, the chemistry of flames may be discussed in terms of both macroscopic and microscopic observations. However, in such gas phase systems, as opposed to the condensed phase, it is possible to rigorously interrelate both macroscopic and microscopic phenomena as suggested in table 13. That is, given the molecular level details of the flame chemistry, one can compute from basic thermodynamic and kinetic principles the macroscopic flame properties.

In dealing with the problem of flame inhibition and extinction a systems approach such as that given in table 14 is suggested. The nature of the experimental, theoretical and basic data requirements is also indicated.

### 3.2. Rating of Flame Inhibitors

The degree of effectiveness of a flame inhibitor can simply be determined from the quantity of the inhibitor needed to extinguish a flame. This quantity will vary with the flame type, i.e., if premixed or diffusion, and the ratio of fuel to oxidant. If the addition of an inhibitor is made to a premixed laminar flow hydrocarbon-oxygen flame, where the reaction zone is luminous, one can determine the effect of the inhibitor, in the absence of extinction, by noting a shift of the reaction zone to a position further downstream from the burner opening. This shift results from a decrease in the burning velocity of the flame due to the presence of the inhibiting agent.

Burning velocity measurements on premixed laminar flow flames provide the main basis for the screening of flame inhibiting agents. Flame inhibition measurements are less frequently made on diffusion flames, due to the difficulty in defining a fundamental flame strength parameter such as burning velocity and also the strong geometrical influences on diffusion flame propagation. However the recent use of an opposed jet burner has provided a means of measuring inhibitor effectiveness in terms of the flow conditions required to produce a hole in the diffusion flame [37].

As the primary purpose of determining the relative efficiency of flame inhibitors relates to their potential application in the area of fire extinguishment and fire prevention, it is pertinent to examine the usefulness of data obtained primarily on model premixed laminar flow flames. Friedman has considered this point on several occasions [10, 13].

The propagation of real, as opposed to laboratory fires, frequently involves diffusion and turbulent flame conditions. However there is evidence for a correspondence between the speed of turbulent flame propagation and the laminar flow burning velocity. Friedman also notes that a diffusion flame does contain a region of premixing at the base and it has not been generally established whether the inhibitor functions in this region or not. It is also found, with CH_3_Br, for example, that a similar amount of inhibitor can extinguish both a diffusion and a premixed flame and this strongly suggests the premixed region to be the relevant area of inhibition [38].

**Figure 5:**
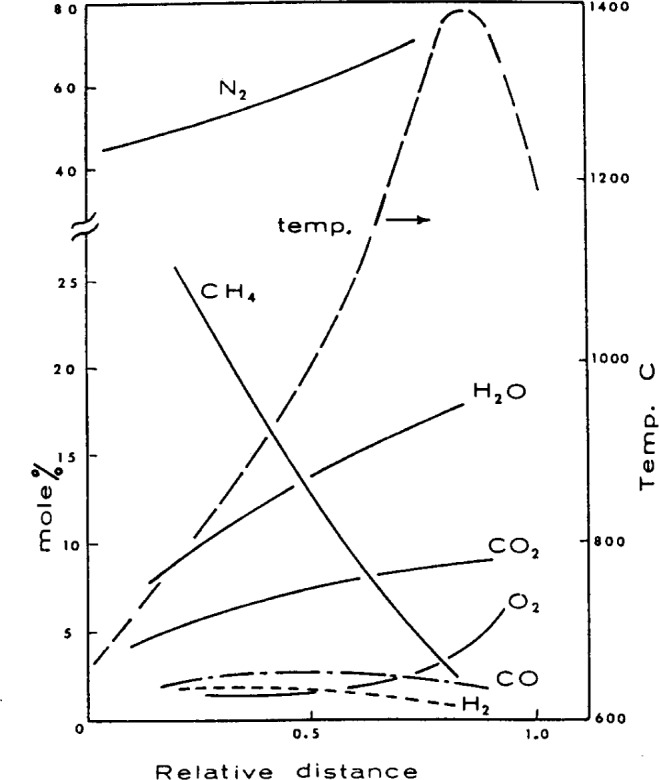
Composition and temperature profiles for a CH_4_–air diffusion flame, taken relative to the center of the CH_4_ fuel column (i.e. zero relative distance) and the CH_4_—air boundary (i.e., 1.0 relative distance).

**Figure 6:**
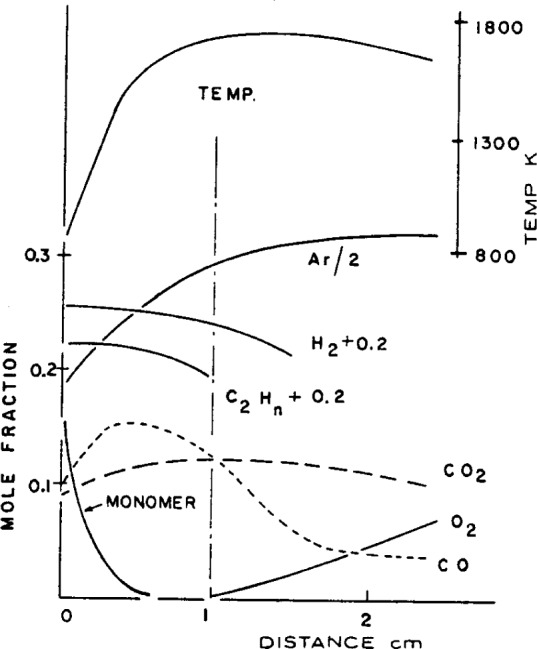
Composition and temperature versus distance above polymer surface profiles for a polymethyl methacrylate candle flame (from Fenimore and Martin, [40]).

One can also argue from the results of flame structure studies on supposedly diffusion flames that such flames resemble fuel rich premixed systems. For example, the chemical structure of a flame generated by passing methane along a tube into the ambient atmosphere, as shown by figure 5, indicates a significant concentration of O_2_ and N_2_ even near the center of the fuel column [39]. Similarly, the preflame region of a polymethylmethacrylate candle flame contains appreciable levels of argon from the surrounding argon-oxygen atmosphere, together with incomplete combustion products such as CO and H_2_ as shown in figure 6 [40].

One can reasonably conclude, therefore, that the measurements of inhibitor reduction of flame speed for premixed flames is an appropriate means of rating potential fire retardants.

As Dixon-Lewis [41] and co-workers have demonstrated, given the basic kinetic and transport properties for the flame reactions, it is possible to quantitatively calculate burning velocities. However, to date this can only be done for the H2–O_2_ flame system, since the basic data and mechanistic understanding are lacking for hydrocarbon and other flames.

Similarly, if the flame kinetics of an additive were known, then it would be possible to predict from basic principles the magnitude of the reduction in flame speed due to inhibition. Since such correlations of flame speed with basic flame data are not feasible at the present time, it is necessary to develop less sophisticated mathematical models for flame inhibition.

The need for such models has recently been emphasized by Fristrom and Sawyer [9], and these authors have developed a model which allows the definition of new parameters for evaluating flame inhibitors in terms of measurable macroscopic variables such as O_2_ concentration, inhibitor concentration and the change in burning velocity. The model has as a basis the notion that the inhibitor sets up a reaction that competes with the chain branching reaction, i.e.:

$$H+O_{2}\to \text{OH}+O$$

by abstracting H atoms, e.g.:

$$H+\text{HCl}\to H_{2}+\text{Cl}.$$

The model follows from the experiments of Wilson, et al., [42] where, in the presence of an inhibitor, the preflame zone is extended and the reaction zone narrows. Inhibition is considered to occur primarily in the preflame region where the chain branching reaction is slow and radical recombination is more important. In this model the inhibitor reduces the level of radicals in the preflame region, but at the reaction zone the concentration may be increased over that for an uninhibited flame. In effect, the radical profiles shift downstream to a higher temperature region, where their competition with the inhibitor is more favorable.

From the mathematical model a figure of merit for an inhibitor, using burning velocity data, may be defined as:

$$\phi_{v}=\frac{\left[O_{2}\right]}{\left[I\right]}\frac{\delta V}{V_{0}},$$

where

*ϕ_v_* is the figure of merit for the inhibitor,
[O_2_] is the initial oxygen concentration,
[*I*] is the initial inhibitor concentration,
*V*_0_ is the burning velocity in the absence of any inhibitor, and
δ*V* is the change in burning velocity due to the presence of the inhibitor.

It is assumed that the flame is slightly fuel rich, i.e., the H atom is the dominant radical.

In practice, *ϕ_v_* correlates with other measures of inhibitor effectiveness, such as blow-off limits and extinction limits for halogen inhibitors. It also follows from the definition of *ϕ_v_* that the degree of inhibition should be proportional to the amount of inhibitor added to the flame. Experimental observations (e.g., see Lask et al. [43]), show that this is approximately valid for a variety of homogeneous inhibitors.

Values of *ϕ_v_* for a selected variety of inhibitor types have been calculated from the data of Lask et al. [43], and unpublished data of Wagner cited by Morrison and Scheller [44] for *n*-hexane fueled flames, and D. Miller et al. [45], for H_2_ fueled flames. The values listed in the table 15 are given in approximate order of increasing effectiveness as flame inhibitors and we shall refer to this table later when discussing specific inhibitor types.

At this point it is sufficient to note that only for the case of CO_2_ is the degree of inhibition explainable in terms of a physical effect such as cooling. The magnitude of *ϕ_v_* greater than unity for the other inhibitors can only be explained in terms of chemical effects. Common commercial extinguishants such as Freons have *ϕ_v_* values similar to the value of 8.4 given for Br_2_. Of the two flame types considered in the table the *n*-hexane/air system more closely resembles the chemical conditions likely to occur in practical fire situations.

### 3.3. Flame Microstructure

In order to provide a molecular basis for flame inhibition it is necessary to identify the elementary reactions involving the flame inhibition and flame propagating radicals. For the ideal case of a premixed laminar flow flat flame it is possible to determine reaction rates and in some cases identify elementary reaction steps. The basic flame relationships are given in table 16. The main experimental input is seen to be the species concentration profiles, i.e., plots of *X_i_* versus z, from which the derivatives are calculated.

Table 17 compares the general capabilities of the two major tools available for determining species concentrations in flames with satisfactory spatial resolution. The present discussion focuses attention on the application of mass spectrometry to flame structure determination and a chronology of the more significant developments in this area is given in table 18.

**Figure 7:**
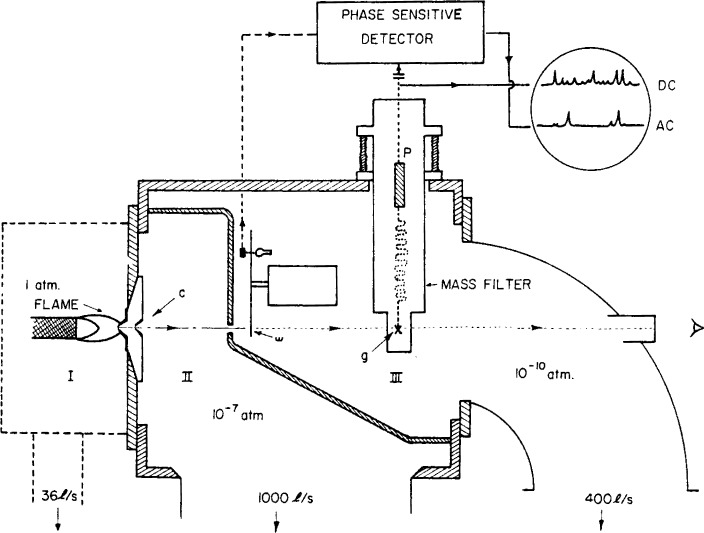
Schematic of mass spectrometric system for sampling I atm flames. A representative gas sample passes through a cone system (c) into an evacuated chamber (region II); the beam then passes through an adjustable orifice into region III, which contains a wheel (w) for mechanical modulation of the beam, an ion source (g) for partial conversion of the molecular species into positive ions, and a mass filter and ion detection system (p), (from Hastie, [46]).

**Figure 8:**
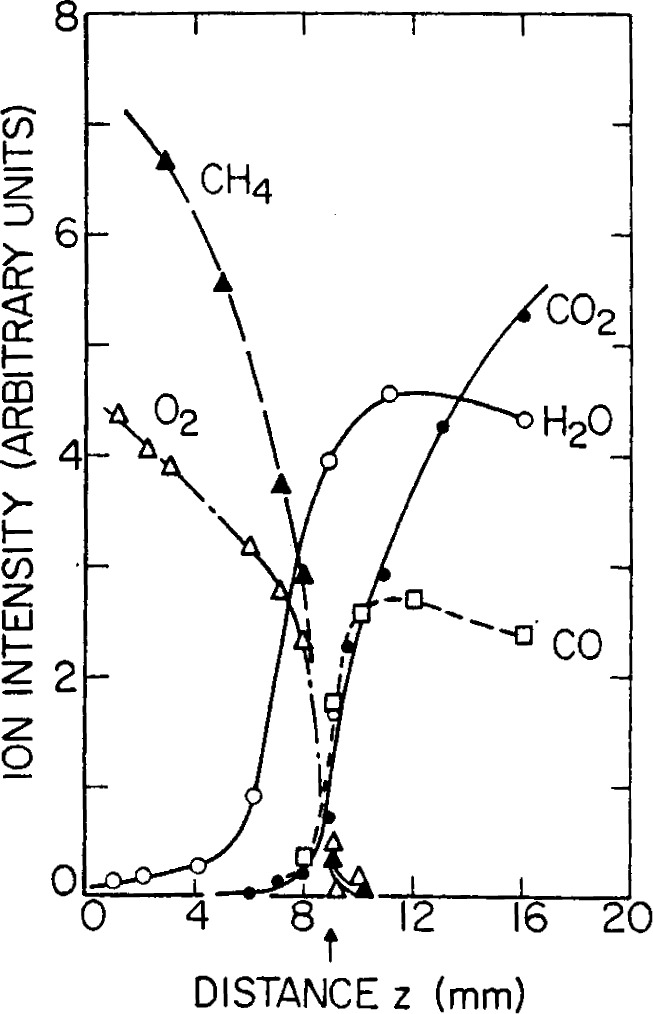
Concentration profiles for major reactants and products in a 1 atm fuel-rich CH_4_-O_2_ flame, (from Hastie, [46]).

**Figure 9:**
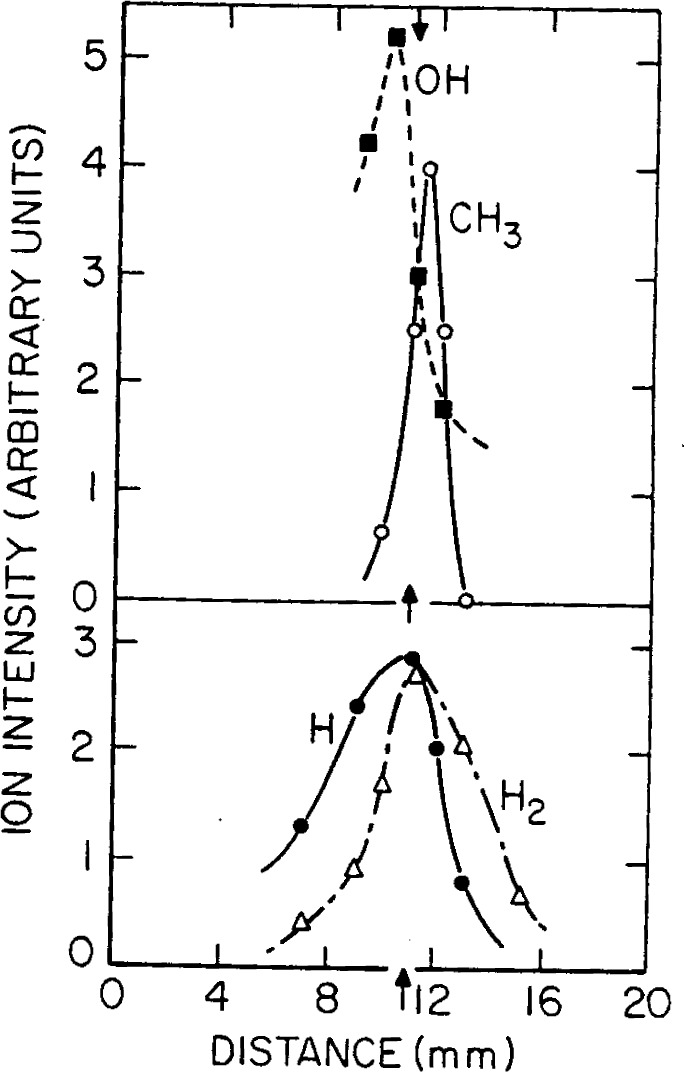
Concentration profiles for the main radical species in a fuel-rich CH_4_-O_2_ flame, (from Hastie, [46]).

Figure 7 shows a schematic of an apparatus used to measure both stable products and reactive intermediates in 1 atm flames at concentration levels down to about 1 ppm [46]. Typical results obtained with this system for CH_4_ fueled flames are given in figures 8 and 9.

### 3.4. Halogen Inhibitors

Halogen systems are among the most widely used commercial reagents for fire prevention and extinguishment. For example, one can cite (a) the early use of CCI_4_ as a portable source of fire extinguishment; (b) the current use of Freons such as CF_3_Br as an extinguishant particularly in connection with fuel fires associated with aircraft mishaps, and also for the protection of electronic equipment; and (c) the incorporation of phosphorus-halogen or antimony-halogen (among others) formulations in materials such as natural and synthetic fabrics, furniture materials, paints, wood, paper, etc., e.g., see Lyons [2].

Halogen systems have also received the most attention by workers interested in the basic mechanism of flame inhibition. It should be recognized that in some instances the halogen treatments serve to alter the course of decomposition of substrates such that only relatively nonflammable gases are produced during the pyrolysis or oxidation processes. However, the present discussion is restricted to the retardancy aspects that involve vapor phase chemistry in the inhibition process.

In practical fire systems the halogen species can be introduced into the gas phase by mechanical means, as with Freon protection systems, or by chemical means, as with the release of HCl from decomposing Polyvinylchloride, or as phosphorus chlorides or oxychlorides formed during decomposition of a polymer substrate, or as antimony halides from polymer substrates.

As is evident from the high *ϕ_v_* values given in table 15 halogen species rank as chemical inhibitors and must therefore interfere with the chemistry associated with flame propagation. Some clues as to the nature of this interference are provided from macroscopic observations such as: (a) the effectiveness of halogens increases in the order fluorides ≪ chlorides < bromides ≤ iodides; (b) CF_3_Br is about four times more effective than CF4 in preventing combustion of n/hexane air mixtures; (c) CF_3_Br is a good inhibitor in hydrocarbon-air flames but is mediocre in H_2_-air flames; (d) Br_2_ has very little effect on CO—O_2_ flames but is effective when small amounts of H_2_ are introduced; and (e) in Br-substituted hydrocarbons and fluorocarbons the inhibitor effectiveness increases with the number of Br atom substitutions and, in certain instances, can be directly proportional to this number. For example, in a stoichiometric methane-air flame, the following *ϕ_v_* values are found: Br_2_ [24], CH_3_Br [12], HBr [11], CF_3_Br [17]. The high value for CF_3_Br seems anomalous and this has been attributed to a possible role of the CF_3_ radical in flame inhibition.

From these macroscopic observations it can be concluded that the flame inhibition must involve an interaction between species containing a halogen and a radical containing a H-atom, such as H or OH.

A more detailed description of the likely flame inhibiting mechanism has been derived from experiments carried out at the molecular level, such as the mass spectrometric microprobe sampling experiments described by Wilson et al. [42], or by Pownall and Simmons [54].

Before discussing the current status of the mechanistic understanding of halogen flame inhibition, it should be noted that a considerable variety of mechanisms have been proposed in recent years. For example, Mills [55] suggested that the inhibiting function of halogen species may be one of electron attachment followed by dissociation to generate active inhibitor species, e.g.:

$$\begin{array}{c} {\text{CF}}_{3}\text{Br}+e\to {\text{Br}}^{-}+{\text{CF}}_{3}\\ {\text{CF}}_{3}+H\to {\text{CF}}_{3}H^{*}\\ {\text{Br}}^{-}+H\to \text{HBr}+e. \end{array}$$

He cites the observation of negative halogen ions in flames as support for this theory. However the concentration of such ions is now known to be at least several orders of magnitude less than for the neutral species and their effect on the flame propagating radicals is negligible.

Creitz [11] suggests that halogenated extinguishing agents act as catalytic agents for the recombination of oxygen atoms. The formation of OX, where *X* may be CI or Br, as an intermediate is suggested; i.e.:

$$\begin{array}{cc} \text{or} & \begin{array}{c} O+X+M\to \text{OX}+M\\ O+X_{2}\;\to \text{OX}+X. \end{array} \end{array}$$

Other workers have suggested that the halogens reduce the concentration of OH in the flame. Rosser et al. [56] observed a decrease in the emission intensity of OH and an increase in that for CH due to the presence of CH_3_Br or HBr in hydrocarbon flames. The observed radiation was not from a very well defined part of the flame, both reaction zone and post flame regions being included. It is well known from flame spectrophotometry studies that emission intensity may not necessarily be correlated directly with radical concentration.

In time resolved studies on the low pressure explosive combustion of styrene-oxygen mixtures, Petrella [57] observed that the production of OH was delayed in the presence of HBr.

The general situation with regard to the action of halogens on OH in flames remains unsettled, and some of the apparent difficulty may be related to the use of different flames for inhibition studies. In particular, Wilson et al. [42] have obtained indirect evidence for an increase in the maximum OH level in low pressure lean CH_4_—O_2_ flames containing HBr. However, using 1 atm lean propane fueled flames, Pownall and Simmons [54] provide indirect evidence for a reduction in the maximum OH concentration in the presence of HBr.

Levy et al. [58] suggest the inhibiting action of HBr to be:

$$\text{HBr}+\text{OH}\to H_{2}O+\text{Br}$$

in lean methane flames. This is analogous to the suggestion of Wilson [59] that methyl bromide reacts as follows:

$${\text{CH}}_{3}\text{Br}+\text{OH}\to {\text{CH}}_{2}\text{Br}+H_{2}O.$$

However, more recently, Wilson and co-workers [42] have suggested an alternative mechanism involving H-atom reactions.

From the recent review of Fristrom and Sawyer [9], the current status of halogen inhibition is as follows. The primary reactions responsible for flame propagation are generally agreed to be:

$$\begin{array}{cc} \text{and} & \begin{array}{c} H+O_{2}=\text{OH}+O\\ O+H_{2}=\text{OH}+H\\ \text{OH}+H_{2}=H_{2}O+H. \end{array} \end{array}$$

In cool flames one must also consider reactions involving HO_2_ such as:

$$\begin{array}{cc} \text{and} & \begin{array}{c} H+O+M\to {\text{HO}}_{2}+M^{*}\\ H+{\text{HO}}_{2}\to \text{OH}+\text{OH}. \end{array} \end{array}$$

From the low pressure flame mass spectrometric studies reported by Wilson et al. [42], it is evident that the introduction of halogen species into a premixed CH_4_/O_2_ flame leads to the production of the hydrogen halide, HX, early in the flame. It was observed that the formation of H_2_CO is inhibited and the production of H_2_ enhanced. This provides indirect evidence for a removal of H atoms from the flame and the predominant reaction is considered to be:

$$H+\text{HBr}\to H_{2}+\text{Br}.$$

This is known to be a fast reaction under flame conditions and Fristrom and Sawyer [9] have demonstrated that it can effectively compete with the chain branching reaction in the preflame region. They also show that the observed degree of inhibition is greater than that which would be predicted by considering the above reaction to reach equilibrium (i.e., to be balanced). The mechanism by which Br is removed from the system has not been established although under relatively cool flame conditions, the reaction:

$${\text{HO}}_{2}+\text{Br}\to \text{HBr}+O_{2}$$

could serve to replenish the inhibitor source. Similarly, the reaction

$$\text{Br}+H+M\to \text{HBr}+M^{*}$$

is possible (Day et al. [60]). The reaction:

X+RH→HX+R

has also been suggested. Such a reaction relies on the presence of fuel (RH) and hence can only function in the preflame mixture.

The production of HBr from other initial sources such as RBr can readily be achieved by reactions of the type:

H+RX→HX+R;

as R is most likely less reactive than H, this reaction is also flame inhibiting.

Note that this inhibition mechanism readily accounts for the noninhibiting properties of fluorides, since the high stability of HF provides an excessively high activation energy barrier for a reaction with H atoms to result. The much lower effectiveness of chloride as compared with bromide inhibitors is probably due to the HCl reaction being very close to thermoneutral, and hence it is likely that the reaction can proceed in the back direction to generate H atoms. The probable importance of the back reaction is evident from the observation that Cl_2_ actually promotes flame propagation in H_2_-air flames, as shown by the negative *ϕ_v_* given in table 15.

This seems to be a very satisfactory model for halogen flame inhibition although it does not account for all of the experimental observations (see Pownall and Simmons [54]). More recent species profile studies also support the model in that H atoms are found to be present in relatively high concentration in the preflame region and in fuel-rich mixtures the radicals O and OH are almost negligible in this region (Hastie [46]). The production of HBr in the preflame region under 1 atm flame conditions has also been established (Hastie [61]).

Another test of the model is provided by the ignition temperature studies of Morrison et al. [44]. The model suggests that the flame reactions are shifted to a higher temperature region and hence the ignition temperature would be expected to be higher in the presence of the halogen inhibitor. Morrison et al. [44] find the expected increase in ignition temperature for methyl halides, SnCl_4_ and BBr_3_. However SiCl_4_, TiCl_4_, CrO_2_Cl_2_ and Fe(CO)_5_ have no effect on the ignition temperature and CCl_4_ actually decreases the ignition temperature. Evidently the model is not of wide generality and it should be noted that the supporting flame microstructure evidence was obtained with 0.05 atm lean CH_4_-O_2_ flames.

In the higher temperature reaction zone region of flames, reactions involving H, OH, and O are known to be balanced and a pseudo-equilibrium exists between these species. Hence under these conditions, arguments as to whether OH, O, or H are the inhibited species are not particularly critical, as a reduction of any of these radicals would serve to inhibit chain branching.

It is known that inhibition is more effective in cooler than in hotter, faster burning flames, and Friedman [13] has suggested that this is due to the higher radical concentrations in the hotter flames. Alternatively, one could argue that in the hotter flames the excess in radical concentration over the equilibrium level is not nearly as great as for cooler flames. Hence from the kinetic, non-equilibrium nature of the inhibition process, one would expect a better degree of inhibition in the cooler flames. The high temperature dissociation of HX for example, could also reduce its effectiveness in the hotter flames.

Flame cooling effects due to thermal dissociation of halogen species, such as Br_2_, are negligible compared to the observed chemical effects (Simmons and Wolfhard [62]). The degree of inhibition observed for Cl_2_ is low enough to be consistent with thermal dissociation as a cooling and slightly flame inhibiting process.

It should be noted that the apparent low effectiveness of CF_3_Br on the fuel side of diffusion flames (Creitz [11]) is explained by Friedman [10] simply as an artificial geometry effect, since the flame itself would be located on the air side of the stagnation point.

### 3.5. Non-Halogen System Inhibitors

It is apparent from the classification of inhibitor effectiveness given in table 15 that non-halogenated compounds such as (CH_3_)_3_PO_4_, Pb(C_2_H_3_)_4_ and Fe(CO)_5_ are one to two orders of magnitude more effective than halogen inhibitors. However systems of this type have not yet been utilized in practice, except that lead tetraethyl has been used to modify the preignition knocking phenomena of internal combustion systems. It is not unreasonable to speculate that the modes of action in flame inhibition and knock prevention may be related. Unfortunately, despite considerable experimental effort, the mechanism of knock inhibition has not been definitively established.

The metal and phosphorus halides indicated in table 15 also show a degree of inhibition which is in considerable excess of what can be accounted for in terms of their halogen content. This is particularly evident in the H_2_-air flame where Cl_2_ itself does not provide any flame inhibition. It is clear that the metals and phosphorus can themselves lead to flame inhibition and, more importantly, to a much greater degree than the halogen inhibitors. The two order of magnitude difference in *ϕ_v_* between Si(CH_3_)_4_ and Pb(C_2_H_5_)_4_ suggests that the inhibition mechanism is particularly sensitive to the properties of the metal itself. In fact, some metals show no inhibition at all. For example, the addition of several percent of Al_2_Cl_6_ vapor to pre-mixed fuel-rich CH_4_/O_2_ flames produced a reduction in burning velocity that could be accounted for entirely from the amount of halogen present [63].

Metals such as Fe, Cr, and Ti, and their oxides, have low vapor pressures under normal flame conditions and their introduction to flames can result in the formation of condensed particles. These particles are highly luminous and are readily observed in the region of the reaction zone and the post flame gases. It has been argued, therefore, that these metals perturb the flame chemistry via heterogeneous rather than homogeneous reactions. A clue to the possible effectiveness of heterogeneous, as opposed to homogeneous inhibition, is provided by the observation of Jost et al. [64], where the flame inhibiting effect of Fe(CO)_5_ reached a limiting upper concentration level. At the higher concentration levels, condensed particles should form and the decreased rate of effectiveness with composition implies that heterogeneous inhibition is less effective than homogeneous reactions. The question of heterogeneous versus homogeneous inhibition has received considerable attention in connection with the function of the commercially used solid extinguishants such as the alkali metal bicarbonates.

#### a. Solid Inhibitors Containing Alkali Metals

It is found that about 10^−3^ mol fraction of powdered alkali metal salts such as K_2_SO_4_, Na_2_CO_3_, KHCO_3_, and NaHCO_3_ can reduce the flame speed of CH_4_-air flames by 50 percent. Thus *ϕ_v_* factors of about 100 are indicated. Powders of alkali metal salts are also found to be more effective on a weight basis than CF_3_Br in a counter flow diffusion flame [37]. Their effectiveness usually follows the order Li < Na < K < Rb. Carbonates are observed to be twice as effective as the alkali metal chlorides.

Flame temperature calculations (Dodding et al. [65]) show that the effect of decomposing NaHCO_3_ on flame cooling is small and that the mode of inhibition must be chemical in nature. In general the smaller the initial particle size of the powder introduced to the flame, the greater the degree of inhibition found (e.g., Dodding et al. [65]; and Rosser et al. [66]).

From these observations, arguments have been given in favor of both solid and vapor phase inhibition mechanisms. Rosser et al. [66] favored a gas phase mechanism, since they calculated that under flame conditions an appreciable vaporization and dissociation of the solid powders should occur. The more recent study of Birchall [68] using town gas-air flames also supports this view. His observations of a relatively high efficiency for the alkali metal oxalates was accounted for by a model where the reactions:

$$\begin{array}{cc} \text{and} & \begin{array}{c} K_{2}C_{2}O_{4}\cdot H_{2}O\left(s\right)\to K_{2}C_{2}O_{4}\left(s\right)+H_{2}O\\ K_{2}C_{2}O_{4}\left(s\right)\to K_{2}{\text{CO}}_{3}\left(s\right)+\text{CO}, \end{array} \end{array}$$

resulted in the production of sub-micron size carbonate particles within the flame. These particles would then readily vaporize and decompose to yield the active inhibitor species. The following order for the effect of the anion on the alkali metal efficiency was indicated: oxide (i.e., oxalate) > cyanate > carbonate > iodide > bromide > chloride > sulfate > phosphate. This order represents the ease with which the alkali metal can be released to form the active species. It should also be noted that the presence of Cl_2_ gas retards the efficiency of the oxalate and cyanate. Also Friedman and Levy [67] have observed that the introduction of elemental Na or K vapor to the fuel side of methane-air counter flow diffusion flames has no effect on the flame strength.

Most workers agree that the active gas phase species would be the hydroxide, KOH. Under equilibrium conditions this is much more stable than the oxides or elemental K. Under very lean flame conditions the oxides may also be important (69). The reaction:

K+OH+M→KOH+M

is considered to be kinetically more favorable than the endothermic process:

$$\text{KOH}+\text{OH}\to H_{2}O+\text{KO}.$$

The reaction:

$$\text{KOH}+H\to H_{2}O+K$$

is considered to be the one responsible for flame inhibition. The poisoning effect of halogens is most likely due to the known stability of the alkali halides in flames resulting in a loss of KOH, for example.

#### b. Other Metal Inhibitors

The refractory nature of the other inhibiting metals and their oxides increases the likelihood of solid formation and a heterogeneous mode of inhibition. Radical recombinations on solid particles should lead to a temperature rise at the particle. For Cr concentrations greater than 10^15^ cm^−3^ in H_2_-O_2_-N_2_ flames, particles were observed which showed a higher temperature than the flame gases, i.e.:

$${\text{Cr}}_{2}O_{3}(s)+H+H\to {\text{Cr}}_{2}O_{3}(s)+H_{2}.$$

However, at lower concentrations it was found that the recombination of H-atoms occured via a homogeneous reaction (Bulewicz and Padley [70]).

In these studies observations were made downstream of the reaction zone and the question arises as to whether the additives have an opportunity to form solids in the preflame region or not. Very little has been done to answer such a question. However, from the light scattering experiments of Cotton and Jenkins [71], it is evident that for the case of Ba at concentrations of 10^−5^–10^−6^ mol fraction in a 1600 K H_2_-O_2_-N_2_ flame, solids do form prior to the reaction zone. These flames did not show the streakiness which is commonly observed when solid particles are present. However, in spite of the presence of solids the observed catalytic effect of alkaline earth metals on the recombination of H-atoms could be explained solely in terms of a homogeneous mechanism.

Indirect evidence for the formation of solids in the pre-reaction zone region of a low pressure CH_4_ flame containing Fe(CO)_5_ additions was reported by Vree et al., [72].

The post reaction zone radical recombination studies made in the presence of metal catalysts, represent the only molecular basis studies that have a bearing on the likely flame inhibiting function of metal containing species. The results of these studies have dispelled the often relied-upon notion that the presence of metallic elements at ppm concentration levels has no effect on flame chemistry.

**Figure 10:**
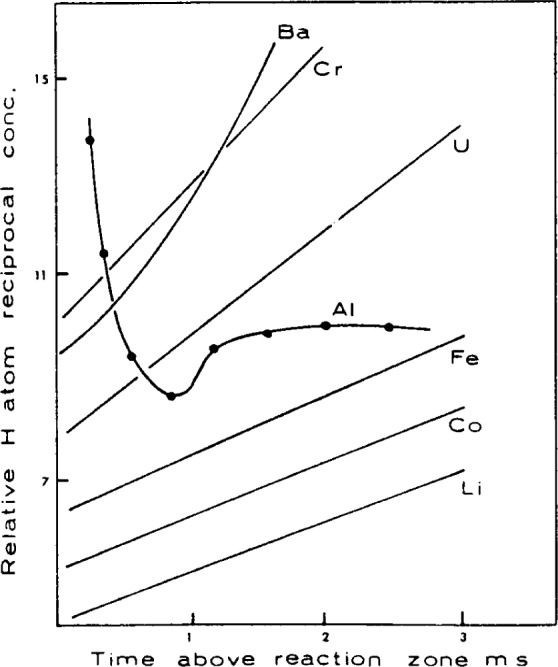
Effect of metal additives on H-atom concentration as a function of time (i.e., distance) relative to the reaction zone in 1 atm H_2_-O_2_-N_2_ fuel-rich flames: curves are vertically displaced for clarity (from Bulewicz and Padley [73]).

**Figure 11:**
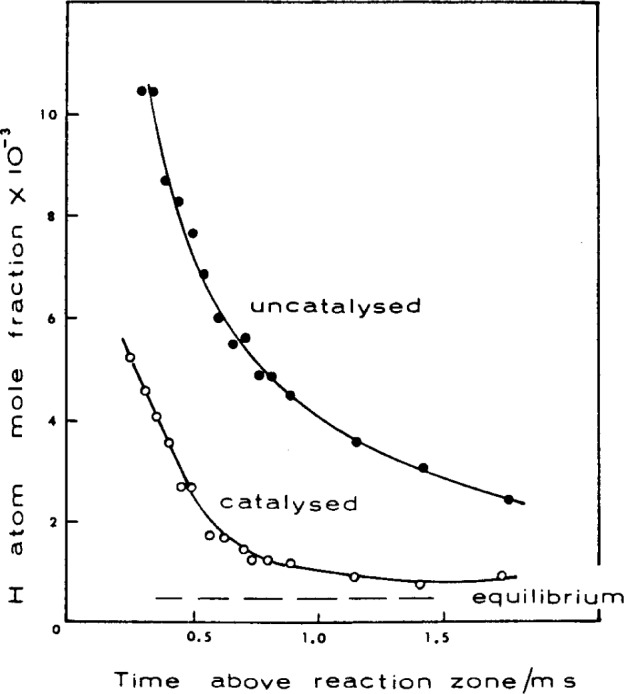
Effect of Cr on H-atom recombination in a 1 atm H_2_-O_2_-N_2_ fuel-rich flame (from Bulewicz and Padley, [70]).

The survey experiments of Bulewicz and Padley [73, 70] as typified by figures 10 and 11, indicate that the elements Mg, Cr, Mn, Sn, U, and Ba had a pronounced effect on the recombination of H atoms, whereas Na, Co, Ni, Cu, V, Zn, Ga, Th, Ce, and La were found to be ineffective as catalysts for H-atom recombination. Some of the results were interpreted in terms of the models given in table 19. That is, the diatomic metal oxide species catalyses the recombination of H or OH radicals via metal hydroxide intermediates.

Many of these oxides and hydroxides are known to be stable under typical flame conditions. Unfortunately, only Sn and Cr are available for comparison with the flame speed measurements represented by table 15. However, both of these elements show high *ϕ_v_* values and they are also among the most effective catalysts for H-atom recombination. Hence it is reasonable to conclude that the role of the metallic flame inhibitors is somewhat analogous to that of the halogens, in that the overall effect results from catalysis of H-atom recombination.

It appears that the oxide-hydroxide mechanism relies on a rather special set of energetics. In particular, MOH must be stable under flame conditions but must readily react with a H-atom when the opportunity arises. Ideally this last step should be exothermic to reduce the probability of a reverse reaction. A more critical set of energetic conditions is revealed by the case of Sn [74], where the initial step involved is,

SnO+H→SnOH*

with SnOH* being produced in an excited electronic state which crosses to another state before undergoing further reaction with another H-atom to produce H_2_ and ground state SnO. Such subtleties in energetics readily account for the stricking variance in the ability of metals to inhibit flames or at least catalyze radical recombination.

The poisoning effect of Cl_2_ on the alkali metal flame inhibition can also be expected to occur for some of the other metal inhibitors. In particular, flame systems containing metals such as Ba, Cr, and Ca, and a halogen source can be expected to form stable MCl and MCl_2_ species. This would reduce the effectiveness of these metals, as they would not be available to participate in the MO—MOH inhibition sequence. On the other hand, metals such as Sb and Sn have relatively weak metal-halogen bonds and the halogen poisoning effect would be minimal in these cases.

### 3.6. Synergistic Systems — The Antimony Oxide-Halogen Example

From the previous discussion of experiments relating to the substrate chemistry involved in the release of halogen and antimony, in the form of SbCl_3_, to the vapor phase it was suggested that the main role of antimony was to moderate the release of halogen to the flame. However, there is evidence to suggest that, given the opportunity to enter the vapor phase, antimony, in the absence of a halogen, can provide a useful degree of flame inhibition. In particular, triphenylstibine shows all the characteristics of an effective vapor phase flame inhibitor [40]. Furthermore, the high value of *ϕ_v_* for SbCl_3_ as compared with Cl_2_ or CCl_4_ (see table 15) also is indicative of antimony, itself, being involved in the flame inhibiting process. In fact, the *ϕ_v_* rating for SbCl_3_ is not very different from that for SnCl_4_ and it is reasonable to consider the possibility of an oxide-hydroxide inhibition process analogous to that suggested earlier for Sn.

**Figure 12:**
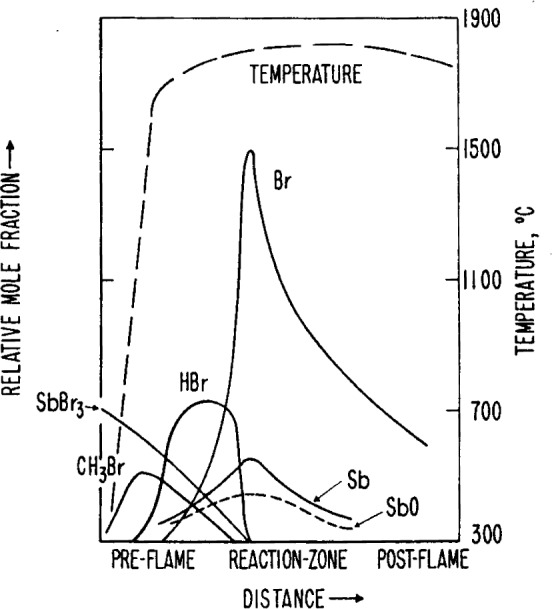
Concentration profiles for species resulting from SbBr_3_ addition to a 1 atm fuel-rich CH_4_-O_2_ flame, (Hastie [61]).

From the results of a recent mass spectrometric analysis of SbCl_3_ and SbBr_3_ in 1 atm fuel rich CH_4_-air flames [61] one can suggest a very plausible mechanism for the role of antimony trihalides as flame inhibitors. The results of the analysis are summarized by the species concentration profiles shown in the figure 12.

The appearance of CH_3_Br and HBr in the preflame region is in accord with the observations of Wilson et al. [42], for halogen additions in low pressure flames. Their model for halogen inhibition also provides the best explanation for this system. The role of antimony as an inhibitor most likely involves the species SbO and Sb which are shown to be the major antimony species in the region of the reaction zone. It should be noted that under the present conditions, i.e., antimony halide mole fractions of 10^−4^ – 10^−3^, no condensation to form solids is possible and heterogeneous mechanisms do not require consideration.

A set of likely reactions involving antimony halides in these flames is given in the table 20. The reactions 4(a)–4(e) are analogous to those suggested for Sn flame inhibition. It would appear from the profile data of figure 12 that inhibition involving SbO species would occur primarily in the region of the reaction zone. This contrasts with the suggested role of HBr where preflame processes are important. Thus we are left with the not unreasonable conclusion that different flame inhibitors may operate in quite different flame regions. Furthermore, as the metal-type inhibitors appear to be more effective than the halogens, it is suggested that inhibition at the reaction zone is more desirable than in the preflame region. It should be noted that the main source of H atoms in the preflame region is from diffusion out of the reaction zone, so that inhibition at this zone could be expected to have a similar delay-effect on the flame reaction to that suggested by the halogen inhibition model.

### 3.7. Phosphorus as a Flame Inhibitor

From the previously discussed studies relating to TPPO-Nylon6-PET substrates, it was concluded that the observed flame inhibition was related to the release of TPPO to the vapor phase, i.e., the flame.

A clue to the possible function of phosphorus additives as flame inhibitors is given by the observations of Fenimore and Jones [75] on low pressure fuel-rich H_2_ flames containing phosphorus. The species HPO was spectroscopically identified in the post combustion gases by its characteristic green chemiluminescence. From indirect mass action-type considerations the species P_2_ was also believed to be a major P-containing species.

**Figure 13:**
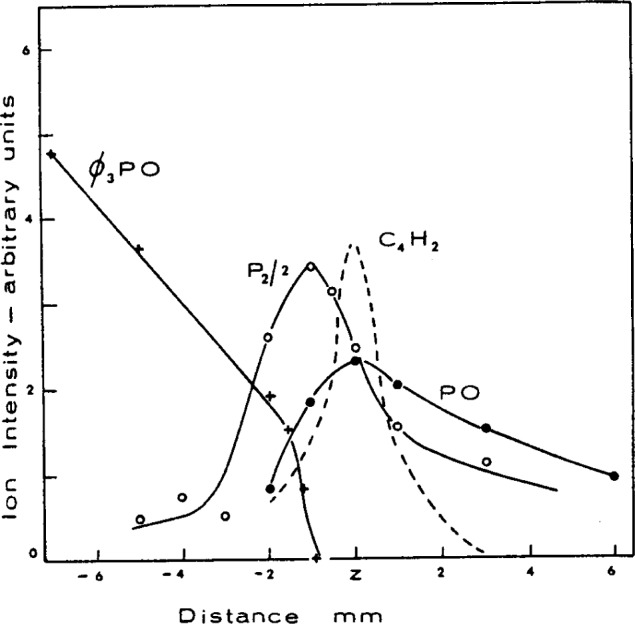
Concentration profiles for species resulting from triphenylphosphine oxide addition to a 1 atm fuel-rich CH_4_-O_2_-Ar flame: distance measurements are relative to the reaction zone at z, (Hastie,[76]).

More recent studies of phosphorus chemistry in flames using the molecular beam sampling mass spectrometric technique have established the nature of the phosphorus intermediate species resulting from the decomposition of TPPO in 1 atm CH_4_- and H_2_-fueled flames [76]. Typical results are given in figure 13. For the fuel rich systems studied, the main species are P_2_, PO, and PO_2_ with lesser amounts of P, HPO, and PN. A catalytic reaction scheme involving these species and leading to H atom recombination is suggested in table 21.

### 3.8. Smoke Suppression

Recent studies involving the mechanism of metal additives on smoke suppression indicate that there appears to be a close relationship to flame inhibition processes.

The suppression of carbon formation in combustion systems can be achieved by (a) adjustment of the operational parameters such as fuel ratio and aerodynamic flow conditions as has been suggested for smoke reduction in jet engines [77]; (b) use of electrical techniques which take advantage of the charged character of flame particulates as has been suggested, for example, by Hardesty and Weinberg [78]; and (c) the use of chemical additives to catalyze the oxidation of carbon within the flame. The use of Ba additives in diesel fuel to suppress exhaust smoke is an example of the chemical inhibitor approach [79].

As was the case with flame inhibition, one is faced with the problem of setting up criteria for defining the degree of effectiveness of additives as smoke reducers Thus Cotton et al. [80] chose the threshold value of the equivalence ratio at which soot was produced for correlation with the degree of effectiveness. Other workers [81] apparently chose a parameter related to the amount of soot produced. As pointed out by Miller [82], these two studies cannot be compared directly and the order of effectiveness of various metals as smoke inhibitors differs considerably.

The smoke inhibition mechanism suggested by Cotton et al. [80] is particularly interesting, as it involves another aspect of the metal oxide-hydroxide catalytic process on H-atoms which was suggested as a mechanism for flame inhibition. It also serves to demonstrate the subtle dependence of the function of flame additives on varying flame conditions.

The survey of Cotton et al. [80], involved the effect of some forty metals on the smoke point of propane-oxygen diffusion flames at atmospheric pressure. These flames in essence had cylindrical symmetry and were non-turbulent. Temperatures were typically in the region of 2100 K. The metals were introduced into the fuel stream as atomized aqueous solutions with N_2_ as a carrier gas.

**Figure 14:**
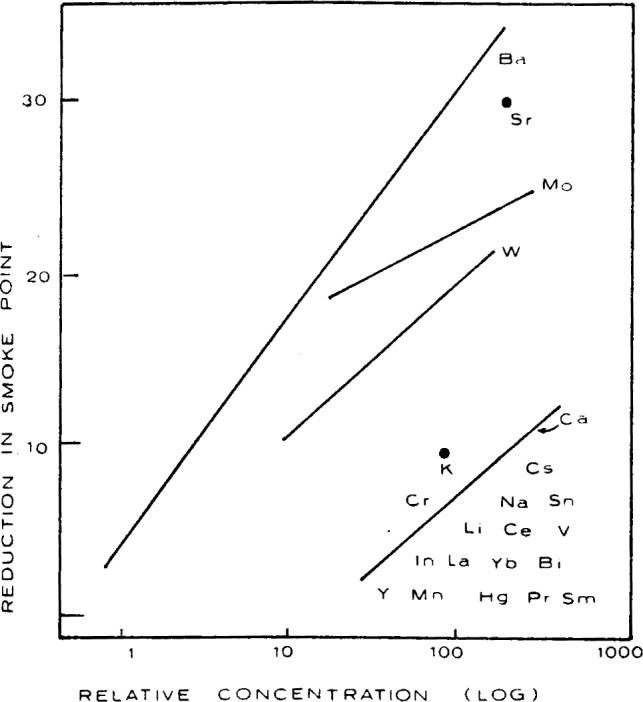
Effect of metal flame additives on smoke reduction relative to that for Ba, (from Cotton, et al., [80]).

The efficiencies of the various metals, relative to Ba, as soot suppressants are represented in the figure 14. The concentration levels of metals were typically in the region of 10^−4^–10^−5^ mol fraction.

The metals not shown in this figure had essentially no effect relative to Ba and they include Ag, Al, As, Be, Cd, Co, Cu, Fe, Mg, Ni, Pb, Si, Th, Ti, Tl, U, and Zn. Notably, several of these, namely Fe, Pb, Si, and Ti, were found to be very good flame inhibitors as discussed elsewhere. From the figure 14, two groups may be distinguished: the group Ba, Sr, Mo, and perhaps W, represent the highly effective smoke inhibitors. Before discussing the significance of these observations, it is pertinent to note that Fe, which has a zero ranking in these studies, is actually used as a commercial smoke inhibitor in the form of ferrocene. Also Spengler et al. [83]. found the addition of Fe in the form of the pentacarbonyl, or as ferrocene, as well as Mn, in the form of methylcyclopentadienyl manganese tricarbonyl, to have a beneficial effect on smoke reduction in several diffusion flames and also a Diesel engine.

A common mechanism was proposed for the soot suppression activity of Ba, Sr, Ca, NO, and SO_2_. From known rate data, the mechanism was demonstrated rather convincingly, at least at a semiquantitative level. Basically the model is as follows. Under the fuel rich conditions necessary for soot production, the active radical concentrations are low and in fact may even be below the equilibrium level. This follows from the well known self-inhibiting effect of hydrocarbons, i.e.,

$$C_{n}+H_{m}+H\to C_{n}H_{m+1}$$

and from the reaction

C(s)+OH→CO+H

which is considered by most workers to be the primary reaction for the oxidation of solid carbon in flames.

The flame condition should be contrasted with that found in faster burning flames, where premixing and less fuel rich conditions exist, with the result that the reaction zone radical levels are well in excess of the equilibrium concentrations. Under these latter conditions Cotton et al. [80], found that Ba and other alkali earth metals catalyzed the recombination of H-atoms. The essence of the smoke suppression mechanism involves a reversal of this recombination process under conditions of low radical concentrations. That is, the metals are involved in a gas phase catalysis of the decomposition of molecular hydrogen or water vapor. The individual steps in this mechanism are given in table 22.

The presence of MO in flames, where M is Ba, Sr or Ca, is well established, as is the formation of the metal hydroxide intermediates. Also the balanced nature of reaction 4 is well established, at least for the hotter flame regions. Thus the net effect of these processes is to increase the formation of OH radicals and hence the oxidation of solid carbon. Note that the reaction 2 leading to the production of the metal dihydroxide is endothermic, and this suggests that the suppression of smoke will be much less effective as the temperature is decreased.

From this model it can be argued that it is not possible to utilize metals for the simultaneous reduction of smoke and flame propagation. This is also suggested by the observation that, as the flames become increasingly more fuel-rich, the catalytic effect on radical recombination is substantially diminished.

Also in real fires, the degree of fuel-oxidant mixing will be an important factor in determining the effectiveness of metallic inhibitors. From arguements such as these, it is not at all surprising that the effectiveness of fire retardant treatments may vary according to the test conditions used. It is also evident that the presence of solid carbon in flames has an inhibiting effect on flame propagation. We may recall that the use of halogens as flame inhibitors often involves an increase in smoke production. Thus smoke production and flame propagation are intimately related phenomena and the use of additives to exclusively suppress one of these, without affecting the other, does not appear to be possible.

### 3.9. Pressure Effects

As much of the experimental evidence for the present understanding of flame inhibition has been derived from molecular studies on low pressure flames, it is important, for scaling purposes, to consider the effect of pressure on inhibition.

Bonne et al. [84] found that as the flame pressure was lowered, the inhibiting action of Fe(CO)_5_ decreased and at low pressure had little effect on the OH concentration or the flame temperature. Inhibition was found to be about a factor of three greater at 1 atm than at half an atmosphere. Similar results were obtained for the effect of trimethyl phosphate (TMP) on the flame inhibition chemistry of H_2_—O_2_—Ar flames [75]. Flames at 0.13 atm pressure were unaffected by additions of 10^−3^ mol fraction TMP; whereas at 1 atm pressure inhibition was observed. A factor of two reduction in the H-atom concentration was also observed when a 10^−3^ mol fraction of TMP was added to the 1 atm flames. Fenimore et al., [75] suggested that the lack of an observable inhibition effect for the low pressure flames may be the result of the much greater relative concentration of H-atoms present in low pressure flames.

More recently, Homann and Poss [85] have found that the degree of inhibition, as represented by a reduction in burning velocity for an ethylene-air flame, is significantly less at 132 torr than at 1 atm for the additives Fe(CO)_5_, CH_3_I, CH_3_Br, and CH_2_Br_2_ but not for CH_2_Cl_2_. In particular, the Fe(CO)_5_ inhibitor was the system most affected by a pressure change. The authors suggest the possibility that this effect is indicative of the role of termolecular reactions, in addition to the normally considered bimolecular steps, in the inhibition process. Such an argument is in keeping with the proposed model for metal oxide inhibition via the hydroxide intermediate.

From these few observations it is clear that one should proceed with caution in attempting to extrapolate the results of inhibition studies on low pressure flames to the “real-life” application of 1 atm systems.

## 4. Conclusions

It is evident that inorganic systems containing halogens (other than F) or certain metals can provide a degree of flame inhibition which is considerably greater than that allowed by physical processes. It also appears that high temperature species such as metal halides, oxides and hydroxides play an important part in the flame inhibition process. As the flame inhibiting processes are kinetically controlled and rely rather stringently on a special set of energetics, the need for basic thermochemical and kinetic data, in support of flame inhibition mechanistic studies, is apparent. Rates of vaporization under pyrolysis and flame conditions also appear to be important quantities in the overall flame inhibition process.

The basic feature of all of the flame inhibiting systems considered is their interaction with H-atoms as the mode of inhibition. In this connection it should be noted that hydrocarbons are themselves strong flame inhibitors. This is particularly evident when hydrocarbons are introduced into H_2_-air flames [45]. The inhibiting effect is thought to be due to reactions such as:

$$H+{\text{CH}}_{4}\to H_{2}{+\text{CH}}_{3}$$

and

$$H+C_{2}H_{4}\to C_{2}H_{5}$$

where the product radicals are far less reactive than the H-atom.

From the flame speed studies of Miller et al., [45] it is evident that the inhibitors are more effective in the more fuel-rich flame mixtures, i.e., at fuel equivalence ratios of greater than about two. Such conditions favor the presence of H-atoms rather than O or OH as the predominant radical species. Hence the suggested inhibition mechanisms, which basically involve catalysis of H-atom recombination, are supported by these macroscopic observations.

It also appears that the inhibition of H atoms can be effectively achieved in both preflame and reaction zone flame regions. The most reasonable model for the inhibiting effect of metals is one involving participation of metal oxide and hydroxide species. This effect is believed to be important primarily in the region of the reaction zone. From the high metal-halogen bond energies for additives such as TiCl_4_ and SiCl_4_ it is most unlikely that any appreciable formation of the monoxide would occur prior to the reaction zone. For such low concentrations of metal additives it is not unreasonable that the onset of condensation and particle formation should lead to a reduction in the rate of inhibition with additive concentration. The formation of particulates would tend to lower the collision probability of radicals with the additive.

As an example of the complexity involved in assessing, from basic principles, the potential of metals as flame inhibitors, consider the Cr system. From the flame speed measurements it is known that Cr has an overall flame inhibiting effect. Similarly the catalytic enhancement of H-atom recombination is also indicative of possible flame inhibition. However, the second stage of hydrocarbon combustion in flames, namely the oxidation of CO to CO_2_ is actually accelerated by the presence of Cr at the ppm concentration level (e.g., see Matsuda et al., [86]). A similar enhancement is found with Ni and Fe additions, but Pb and Te show a retarding effect on this oxidation.

It is apparent that the various suggested mechanisms do not satisfy all of the macroscopic observations and general questions arise. For example, why do PCl_3_ and PBr_3_ have the same effectiveness in *n*-hexane-air mixtures when Br_2_ and HBr are substantially superior to Cl_2_ or HCl? Why is the inhibiting action of halogens more pronounced for fuels with low hydrogen content (e.g., C_6_H_6_) than for H_2_ or CH_4_ [43]? Why is Fe(CO)_5_ much less effective in H_2_-fueled flames? Similarly, why is Fe(CO)_5_ less effective in an O_2_-CH_4_ flame than an air-CH_4_ stoichiometric flame [43]?

Answers to such questions and a definitive understanding of the mechanisms involved in flame inhibition can only result from molecular level studies of flame reaction kinetics.

## Figures and Tables

**Table 1:** 

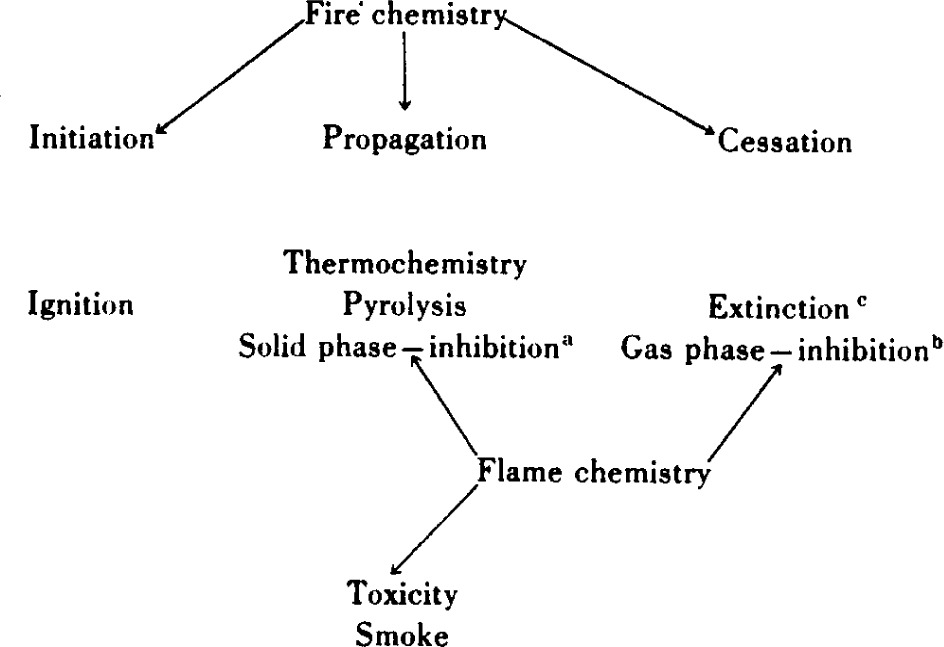

a Production of noncombustibles on pyrolysis.

b Pyrolysis accompanied by in situ release of flame poisons.

c External introduction of flame poisons.

**Table 2:** Microscopic view of a burning system

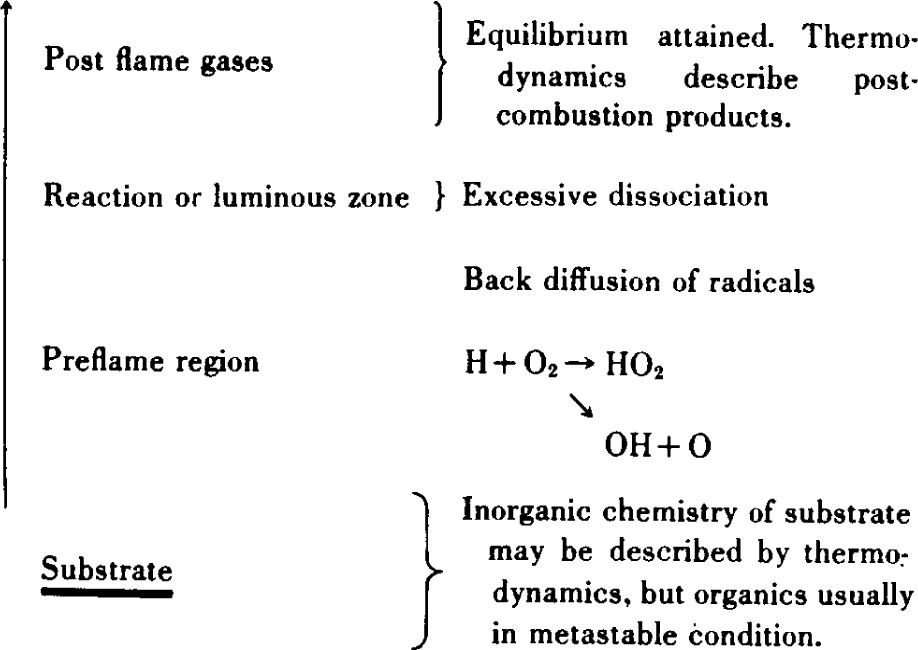

**Table 3:** Macroscopic criteria of chemical mechanism

Gas phase:
Loss of retardant element from substrate
Insensitivity to structure
Sensitivity to oxidant
No change in composition of volatiles
Solid phase:
Enhanced char formation
Retention of retardant element in substrate
Retardant element ineffective in gas phase
Retardancy activity sensitive to structure of substrate
Retardancy insensitive to oxidant, e.g., O_2_ or N_2_O
Change in composition of volatile pyrolysis products in presence of retardant

**Table 4:** Antimony oxide — halogen system as a model study

A. Widespread use
e.g. in both natural and synthetic fibers, plastics, wood, paper, paint, etc.
B. Is synergistic
e.g. 3% Sb_4_O_6_ + 5% Br ≡ 15% Br
C. Has gross characteristics of a gas phase process
e.g. antimony and halogen lost on pyrolysis
D. Exists a need for substitute systems due to a marked fluctuation in availability and price of Sb_2_O_3_

**Table 5:** Macroscopic observations relating to antimony-halogen action

Cotton — Sb_2_O_3_—plasticized PVC
1. Treated material chars at 280 °C; untreated fabric relatively unaffected
2. 80% of Sb vaporized
(Read and Heighway-Bury [24])
Chlorinated polyethylene—Sb_2_O_3_
1. Loss of Sb in vapor when chlorine present
2. Sb_2_G_3_ ineffective in absence of Cl
3. L.O.I, shows effect in O_2_—N_2_ (chain branching), not in N_2_O—N_2_ (nonchain branching)
4. Composition of volatile gases independent of presence of Sb_2_O_3_
(Fenimore and Jones [27])
Sb-Halogen system reduces crack growth, facilitates homogeneous char development in unsaturated polyester resins
(Einhorn [3])
Ignition behavior of polyester resins suggests gas-phase inhibition
(Learmonth, et al. [19])

L.O.I. is a common abbreviation for the limiting oxygen index flame retardancy test, e.g., see ref. [27].

**Table 6:** Molecular level observations in the Sb_2_O_3_—HCl system

1. HCl reacts rapidly with Sb_2_O_3_ solid at *T* ≃ 240–460 °C to yield SbCl_3_ and H_2_O as the only vapor species.
2. Solid SbOCl forms at *T ≃* 200–250 °C.
3. Solid SbOCl decomposes to yield SbCl_3_ over the range *T ≃* 250–450 °C.
4. Solid Sb_2_O_3_, in the absence of halogens, does not vaporize over the typical pyrolysis temperature regime of 300–450 °C.

**Table 7:** Antimony oxide —halogen substrate reactions

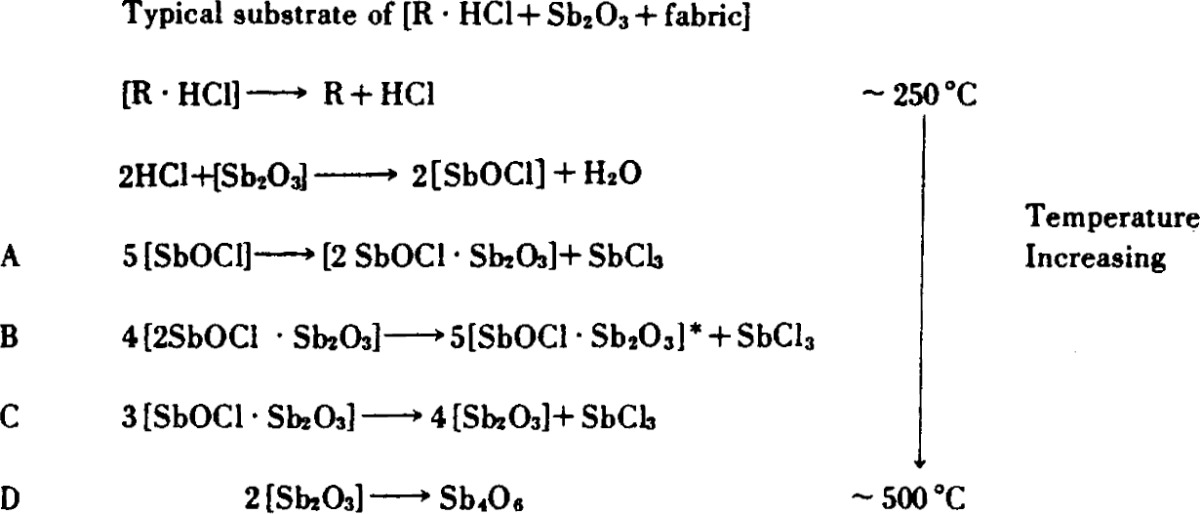

Square brackets denote solids.

* Could also be [2SbOCl · 3Sb_2_O_3_], i.e., see [28].

**Table 8:** Effect of triphenylphosphine oxide (TPPO) on flammability of polyester^a^

Pure polyester	Burns
Polyester + 1 *mol %* TPPO	Self extinguishing in 3–8 s
Polyester + TPPO + Nylon-6 (1% each)	Nonburning or self extinguishing in 0–3 s

a From Bostic and Barker [35].

**Table 9:** Reactions leading to the release of TPPO to the vapor phase^a^

Reaction	Temperature	
[TPPO]	→ TPPO (g)	90 °C
Complex A [TPPO · PET]	→ TPPO	160 °C
Complex B [TPPO · PET]	→ TPPO	200–240 °C
Complex B^1^ [TPPO · Ny6 · PET]	→ TPPO	220–320 °C
Complex C [TPPO · Ny6]	→ TPPO	130–210 °C

a Square brackets denote condensed phase.

**Table 10:** Molecular nature of 1 atm flames

Species-type	Examples	Typical concentration (mole fraction)
		
Stable reactants and products..	CH_4_, O_2_ CO_2_, H_2_O	0.99 total
Intermediates...............	C_2_H_2_, CH_3_CHO, C_4_H_2_	10^−2^ 10^−4^
Radicals...............	H, OH, O, CH_3_ HO_2_, C_2_H	10^−2^ 10^−4^
Positive ions...............	H_3_O^+^, CHO^+^	≤10^−5^
Negative ions...............	OH^−^, Cl^−^	≤10^−7^

**Table 11:** Time scale in 1 atm flames (T ~ 2000 K)

Gas velocity	= 10^2^–10^3^ cm s^−1^
Reaction zone thickness	= 10^−2^ cm
Residence time – at reaction zone	= 10^−4^ – 10^−5^ s
Chemical reaction – bimolecular	⩽ 10^−5^ s (for 20–30 k cal mol^−1^ activation energy)
Relaxation of excited species	
–Translational	= 10^−8^ s
–Rotational	= 10^−8^–10^−7^ s
–Vibrational	= 10^−6^– 10^−3^ (10^−4^ typical) s
Electronic – chemiluminescence	⩾ 10^−3^ s
Diffusion velocity	
– H atoms	= 100 cm s^−1^
– O atoms	= 10 cm s^−1^

**Table 12:** Flame reaction mechanisms

Rich H_2_−O_2_−N_2_^a^:	
	OH+H_2_ → H_2_O + H
	H+O_2_ → OH+O
	O+H_2_ → OH + H
	H+H+M → H_2_ + M
	H+H_2_O → OH+H_2_
	O + H_2_O → 2OH
	H+OH+M → H_2_O+M
	H+OH+H_2_O→2H_2_O
	H+O_2_+M→HO_2_+M
	H_2_ + HO_2_→H_2_O_2_ + H
	H_2_O_2_ + M→2OH+M
	H+HO_2_→2OH
	H+H_2_O_2_→H_2_O+OH
	OH+H_2_O_2_→H_2_O+HO_2_
	2HO_2_→H_2_O_2_+O_2_
Hot-rich CH_4_−O_2_:	CH_4_+H=H_2_ + CH_3_
Hot-lean CH_4_−O_2_:	CH_4_ + OH = H_2_O + CH_3_
Cool CH_4_−O_2_:	CH_4_ + O_2_ = HO_2_ + CH_3_

a e.g., see Wilde, K. A., Combust. Flame **18**, 43 (1972).

**Table 13:** Flame chemistry— tools and techniques

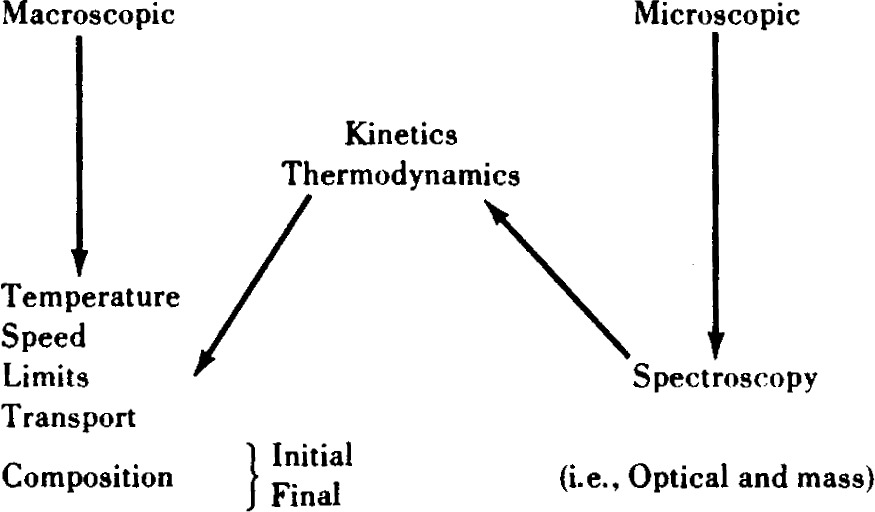

**Table 14:** Flame inhibition and extinction—systems approach

Programs	Classification
	
(a) Identify flame species	Spectroscopy
(b) Determine flame kinetics	Basic data
(c) Test kinetic models	Theory – flame equations
(d) Determine optimum flame species for inhibition	Basic data
(e) Design stable molecular precursors to inhibitor species	Thermodynamics, kinetics basic data
(f) Determine solid sources of such molecular precursors	Thermochemistry, basic data
(g) Define chemistry of incorporation of additives to polymer substrates	Thermochemistry, solid state – structural studies

**Table 15:** Relative effectiveness, ϕ_ν_, of selected flame inhibitors

Inhibitor^c^	Flame type	
	*n*-hexane/air^a^ *ϕ_ν_*	H_2_-air^b^ *ϕ_ν_*
CO_2_	0.86	
Cl_2_	1.8	d−0.26
Si(CH_3_)4	3.9	
CCl_4_	4.2	
Br_2_	8.4	
SiCl_4_	10.5	3.5
(CH_3_)_3_PO_4_	23	
SbCl_3_	26	
TiCl_4_	30	10
SnCl_4_	31	12.9
POCl_3_	31	7.2
PCl_3_	39	4.5
PBr_3_	39	
CrO_2_Cl_2_	⪎244	
Fe(CO)_5_	356	19
Pb(C_2_H_s_)_4_	390	

a From data given by Lask et al. [43], for a stoichiometric mixture.

b From data given by D. Miller et al. [45], for a mixture with 1.75 fuel equivalence ratio.

c Amounts of inhibitor used varied from 0.015 percent to several volume percent.

d Negative sign indicates flame speed increase rather than decrease.

**Table 16:** Some basic flame relationships^a^

Diffusion velocity (normal to flame front)
$Vi=-\frac{dXi}{dz}\left(\frac{Di}{Xi}\right)$
Fractional mass flow
$Gi=Xi\frac{Mi}{\bar{M}}\left(\frac{v+Vi}{v}\right)$
Net reaction rate
$\frac{dXi}{dt}=\frac{dGi}{dz}\left(\frac{\rho v}{Mi}\right)$
Reaction mechanism e.g., for
$i+l\to j+m,-\frac{dXi}{dt}=\frac{dXj}{dt}=k\left[Xi\right]\left[X_{l}\right]$
Mass balance
$\sum_{i}\frac{n_{i}G_{i}}{Mi}=\text{const}.$
Notation
*X_i_* = mole fraction of species *i*
*Vi* = diffusion velocity
*ν* =gas velocity
*ρ* = density
*z* = distance
*D* = diffusion coefficient
*M* = molecular weight
*n_i_* = number of atoms of an element in species *i*
*k* = reaction rate constant

a See e.g., Fristrom, R. M., and Westenburg, A. A., Flame Structure, (McGraw-Hill Co., New York, 1965).

**Table 17:** Tools for concentration profile measurements – general capabilities

Optical spectroscopy	Mass spectroscopy
	
Detection of most atoms and diatomic species	Detection of most species
OH radical	All radicals e.g., H, OH, O, CH_3_, HO_2_
Detects excited states	—
Combustion products – difficult to resolve	Good capability
Absolute concentration – some difficulty in both cases	
Fair spatial resolution	Good spatial resolution
No perturbation of system	Some perturbation by probes

**Table 18:** Chronology of flame mass spectrometry for – uncharged species

Author	Reference	Year	Technique
			
Eltenton...............	[47]	1947	Low pressure flame (L. P.), radicals ? flame modulation.
Foner & Hudson.......	[48]	1953	L. P. radicals molec. beam modul.
Bryce, et al...............	[49]	1956	L. P., radicals ? no modulation.
Fristrom, et al..........	[50]	1961–1963	L. P., microprobe.
Fenimore, et al.........	[51]		
Dixon Lewis..........	[52]	1963	1 Atm., stables no modul.
Milne & Greene	[53]	1966	1 Atm., radicals, mol. beam modul.

**Table 19:** Mechanisms for H-atom recombination^a^

Sn:	
SnO + H+X	→SnOH+X
SnOH + H	→SnO + H_2_
M = Ca, Sr, Ba:	
MOH + H	→MO + H_2_
MO + H_2_O(+X)	→M(OH)_2_(+X)
M(OH)_2_ + H	→MOH + H_2_O

a See refs: [71], [73], [74].

**Table 20:** Reactions involving antimony trihalides in CH_4_-O_2_ flames^a^

1.	(a)	SbX_3_ + H→HX+SbX_2_
	(b)	SbX_3_→SbX_3_*→X + SbX_2_
	(c)	SbX_3_ + CH_3_→CH_3_X+SbX_2_
2.	(a)	SbX_2_+H→HX+SbX
	(b)	SbX_2_ + CH_3_→CH_3_X+SbX
3.	(a)	SbX+H→Sb+HX
	(b)	SbX + CH_3_→Sb+CH_3_X
4.	(a)	Sb+O+M→SbO+M^*^
	(b)	Sb+OH+M→SbOH+M^*^
**	(c)	SbOH+H⇌SbO+H_2_
**	(d)	SbO+H→SbOH^*^
	(e)	Sb + H_2_O⇌SbO+H_2_
5.	(a)	X + X+M→X_2_+M^*^
	(b)	X_2_ + CH_3_→CH_3_X + X
	(c)	X+CH_3_ + M→CH_3_X+M^*^
**6.	(a)	HX+H→H_2_ + X
	(b)	HX+CH_3_→CH_4_ + X
7.	(a)	X + HO_2_→HX+O_2_
8.	(a)	CH_3_X+H_2_→CH_4_+HX
	(b)	CH_3_X+H→CH_4_ + X or CH_3_+HBr
	(c)	CH_3_X→CH_3_X^*^→CH_3_ + X

** Reactions leading directly to flame inhibition.

a See also ref. [61].

**Table 21:** Probable reactions leading to inhibition in flames containing phosphorus

(C_6_H_5_)_3_PO	**→**	PO, P, and P_2_
H+PO+M	**→**	HPO + M
OH+PO	=	HPO+O
HPO + H	=	H_2_ + PO
Other likely reactions		
P_2_ + O	=	P + PO
P+OH	=	PO + H

**Table 22:** Mechanism for catalytic oxidation of smoke^a^

MO + H_2_	**→**	MOH + H	[1]
MOH + H_2_O	***→***	M(OH)_2_+H	[2]
M(OH)_2_+(X)	**→**	MO + H_2_O + (X)	[3]
H + H_2_O	=	OH + H_2_	[4]
OH+C(s)	=	CO + H	

a From Cotton et al., [80].

Note: H is initially below equilibrium concentration.

## Footnotes

1 Figures in brackets indicate the literature references at the end of this paper.
